# The benefits of exercise on aging: focus on muscle biomarkers

**DOI:** 10.18632/aging.206064

**Published:** 2024-08-08

**Authors:** Robin Grolaux, Bernadette Jones-Freeman, Macsue Jacques, Nir Eynon

**Affiliations:** 1 Australian Regenerative Medicine Institute, Monash University, Melbourne, Australia

**Keywords:** skeletal muscle, exercise, epigenetics, OMICs, biomarkers

## Abstract

The focus on maintaining health and vitality (e.g., good healthspan) in later life has become increasingly important as the world’s population is getting older. In the last decade, advances in aging research have identified biomarkers like DNA methylation (DNAm) and gene expression, offering insights into both chronological and biological aging. This understanding opens up possibilities for interventions that can slow down molecular aspects of the aging process. Exploring the impact of exercise on these biomarkers in human skeletal muscle (a critical tissue for metabolism, thermogenesis and movement) reveals its potential to foster healthier aging.

## What we know

Over the past few decades, groundbreaking research in the field of aging has deepened our understanding of the molecular basis of this process. Now, we can predict someone’s chronological age based on a handful of biomarkers that reflect the state of various molecular processes [1]. More strikingly, those markers are also associated, to some extent, with a person’s biological decline, known as biological age. Among those biomarkers, DNAm and gene expression have been extensively studied.

The correlation between biomarkers and aged-related biological decline has led to the idea that aging could be slowed down, if not reversed, known as the geroscience hypothesis. This theory suggests that we could use predictive biomarkers to quantify the impact of interventions aimed at improving healthspan. Research has largely focused on identifying those biomarkers in blood, one of the most accessible tissues [2]. However, age-related conditions can be highly tissue-specific, and therefore such should be interventional therapies aimed at improving health in later life [3]. Furthermore, aging is a heterogeneous process that affects individuals differently, making the size of a clinical study critical for detecting significant changes.

## What we’re learning

In a large cohort of human skeletal muscle samples, our group previously highlighted the effects of aging in this tissue and developed the Muscle Epigenetic Age Test (MEAT), a highly accurate predictor of chronological age based on DNAm biomarkers [4]. Since DNAm only provides part of the picture, subsequent research integrated transcriptomics and proteomics data to comprehensively analyze aging across different omics [5]. In our recent paper “Exercise is associated with younger methylome and transcriptome profiles in human skeletal muscle”, published in the May 2023 issue of Aging Cell [6], we went further and investigated the effect of exercising on age-associated biomarkers. By leveraging the statistical power of meta-analyses on 37 datasets representing 3176 human muscle samples, we identified Differentially Methylated Positions (DMPs) and Differentially Expressed Genes (DEGs) associated with aging in human skeletal muscle. Then, we assessed if those biomarkers of aging showed a significant association with baseline fitness levels, muscle disuse, and exercise interventions.

Our findings revealed that muscle disuse significantly accelerates transcriptomic aging (i.e., Transcriptomic profiles of individuals with muscle disuse were similar to older people). Moreover, we demonstrated that maximal oxygen consumption or VO_2max_, the gold standard measure of fitness, and exercise interventions are associated with transcriptome and methylome levels comparable to the ones of younger individuals. This suggests an inverse relationship between fitness status and aging, underscoring the benefits of exercising for more youthful transcriptomes and epigenomes.

At the physiological level, aging directly impacts human skeletal muscle, leading to a loss of muscle mass and function, which results in adverse outcomes. Our previous studies showed these effects are also evident at the methylome, transcriptome, and proteome levels [5]. Exercise, a highly accessible therapy, has already demonstrated a plethora of health benefits, including mitigating age-related declines.

Here [6], we presented the first quantitative and qualitative study on the effect of exercise on age-related biomarkers in human skeletal tissues. Despite the large number of samples, our study was still underpowered to capture all the impact of exercise on age-related DMPs of DEGs, this could be explained by the heterogeneous protocols used in the different studies we analyzed Nevertheless, our robust meta-analysis approach is highly scalable, and we are confident that applying it to more cohorts will yield further insights.

## Where we’re going

Future research could focus on the global effects of exercise on various molecular pathways and differentiate between the types of exercise to develop more efficient personalized therapies. This is a direction our group, and others, have chosen to explore, as discussed elsewhere [7]. Additionally, studies should shift their focus away from correlation to causality, necessitating carefully designed longitudinal studies to avoid batch effects typically occurring in cross-sectional studies. Mendelian Randomization analyses have proven to be an efficient statistical method to infer causality and discriminate between adaptive and damaging methylation changes [8]. However, both genetic and epigenetic profiles are necessary for their implementation. Most of the studies on aging focus on DMPs, but we and others [3, 9, 10] have shown that Variably Methylated Positions (VMPs) are also correlated to aging and their systematic inclusions in age-focused analyses would help shed light on their potential role in this process. These are exciting times. Research on age-related biomarkers has opened the possibility of validating the molecular impacts of health-promoting therapies. Open science enables us to interrogate biological data with ever-increasing statistical power. Collecting FAIR (Findable, Accessible, Interoperable, Reusable) data from large and inclusive cohorts will be key for detecting small biological effects.

We have the opportunity to uncover functional therapies that effectively impact aging. While there is still a long way to go, these initial steps pave the way toward a healthier lifespan.

## References

[1] Moqri M, et al. Nat Med. 2024; 30:360–72. 10.1038/s41591-023-02784-9 38355974 PMC11090477

[2] Lu AT, et al. Aging (Albany NY). 2019; 11:303–27. 10.18632/aging.101684 30669119 PMC6366976

[3] Seale K, et al. Nat Rev Genet. 2022; 23:585–605. 10.1038/s41576-022-00477-6 35501397

[4] Voisin S, et al. J Cachexia Sarcopenia Muscle. 2020; 11:887–98. 10.1002/jcsm.12556 32067420 PMC7432573

[5] Voisin S, et al. J Cachexia Sarcopenia Muscle. 2021; 12:1064–78. 10.1002/jcsm.12741 34196129 PMC8350206

[6] Voisin S, et al. Aging Cell. 2024; 23:e13859. 10.1111/acel.13859 37128843 PMC10776126

[7] Jacques M, et al. bioRxiv. 2024. 10.1101/2024.07.14.603458

[8] Ying K, et al. Nat Aging. 2024; 4:231–46. 10.1038/s43587-023-00557-0 38243142 PMC11070280

[9] Seale K, et al. bioRxiv. 2023. 10.1101/2023.12.20.572666

[10] Slieker RC, et al. Genome Biol. 2016; 17:191. 10.1186/s13059-016-1053-6 27654999 PMC5032245

